# Therapeutic Challenges and New Era in Fibrillary Glomerulonephritis with the Introduction of DNAJB9: Experience from a Tertiary Nephrology Center

**DOI:** 10.3390/jcm14113709

**Published:** 2025-05-26

**Authors:** Tsielestina Poulli, Paraskevi Liaveri, George Liapis, Maria Daoudaki, Ariadni Fouza, Maria Stangou, George Moustakas

**Affiliations:** 1 Department of Nephrology, General Hospital of Athens “Georgios Gennimatas”, 154 Mesogeion Avenue, 11527 Athens, Greece; chelestinapoulli@outlook.com (T.P.); paraskeviliaveri@yahoo.com (P.L.); georgemoustakas@gmail.com (G.M.); 2 Department of Pathology, School of Medicine, National and Kapodistrian University of Athens, 11527 Athens, Greece; gliapis@gmail.com; 3 Laboratory of Biological Chemistry, School of Medicine, Faculty of Health Sciences, University Campus, Aristotle University of Thessaloniki, 54124 Thessaloniki, Greece; ariadnefou@gmail.com; 4 1st Nephrology Clinic, Hippokration General Hospital of Thessaloniki, School of Medicine, Faculty of Health Sciences, Aristotle University of Thessaloniki, 54642 Thessaloniki, Greece; mstangou@auth.gr

**Keywords:** DNAJB9, fibrillary glomerulonephritis, kidney biopsy

## Abstract

**Background/Aim:** Fibrillary glomerulonephritis (FGN) is a rare glomerular disease characterized by non-amyloid fibrillary deposits in the glomeruli and positive staining for DNAJB9. There is currently no treatment of choice, and the poor prognosis highlights the need for further research. We aimed to investigate the clinical and pathological characteristics and outcomes of FGN patients from a tertiary nephrology center. **Methods:** A retrospective cohort study of eleven patients diagnosed with FGN between 2016 and 2025, based on kidney biopsy and DNAJB9 positivity, was used. Partial response was defined as a ≥50% reduction in proteinuria with stable renal function. **Results:** At diagnosis, nine patients had nephrotic-range proteinuria, and eight had microscopic hematuria. Mean serum creatinine was 1.6 mg/dL, and mean proteinuria was 3.78 g/24 h. Comorbidities included SLE (n = 1), sarcoidosis (n = 1), and lung cancer (n = 1). The most common histological pattern was mesangial proliferative (n = 6). DNAJB9 staining was positive in five patients. All patients received RAAS blockade and immunosuppression (e.g., corticosteroids, rituximab). Partial response occurred in 73% with a median follow-up of 24 months, with 80% showing >50% proteinuria reduction. One patient died during follow-up; no patients progressed to ESRD or required dialysis. **Conclusions:** FGN is clinically diverse and lacks a standard treatment. The small sample size limits generalizability.

## 1. Introduction

The term “fibrillary glomerulonephritis” (FGN) was first introduced in 1987 by Alpers et al. [1] to describe a distinct entity of unknown etiology. It was defined by the presence of randomly arranged non-amyloid fibrillar deposits 14–24 nm in diameter in the glomeruli, negative for Congo red staining, and positive by immunofluorescence for IgG, C3, and both kappa and lambda light chains [2]. FGN has been observed in 0.4–1.4% [3,4] of kidney biopsies, predominantly affecting individuals aged 49–60 years, and is more common in white women [3,5].

FGN has been associated with a number of diseases, including autoimmune diseases, cancers and viral infections. The strongest links are with autoimmune diseases [3], affecting 13–30% of patients, followed by cancer (4–23%) [6], especially solid organ tumors. Malignancy may precede or follow the diagnosis of FGN, and cases where FGN remission occurs with malignancy treatment are rare [6]. FGN has been linked to hepatitis C (10% to 27% in non-whites) [7], and hepatitis B is rare [8]. Finally, there is a link between FGN and HIV, especially among those with hepatitis C [9].

FGN is characterized by the manifestation of nephrotic syndrome (characterized by proteinuria, hematuria, renal insufficiency, and hypertension) with rapidly progressive glomerulonephritis due to a crescentic phenotype in some patients [3,7]. Serum cryoglobulins and rheumatoid factor are often negative, and hypocomplementemia is rare. Hypogammaglobulinemia is usually absent, and serum IgG subclass levels are normal [3,7].

FGN typically presents with six distinct histological patterns on light microscopy. These patterns correlate with clinical features and prognosis and include (a) mesangial proliferative, (b) membranoproliferative, (c) endocapillary hyperplasia, (d) crescentic, (e) membranous, and (f) diffuse sclerosis patterns. Mesangial proliferative is the most common, occurring in 21–78% of cases [5,10,11].

While these histological patterns provide important diagnostic clues, they are not specific to FGN. A major breakthrough in improving diagnostic specificity came in 2018, when two independent research teams from the Mayo Clinic and the University of Washington discovered the presence of DNAJB9 at high levels in the glomerular proteome [12,13,14]. The Mayo Clinic study showed that DNAJB9 was present in all 24 cases of FGN; whereas, its absence was observed in 145 cases of amyloidosis, 72 cases of other GN, and the 12 healthy controls. The co-localization of DNAJB9 and IgG was also found in the mesangium and glomerular basement membrane, with DNAJB9 also found in FGN fibrils but not in fibrils associated with amyloidosis or immunotactoid GN [13,14]. Similar results were documented by the University of Washington, where DNAJB9 was identified in all 11 glomeruli of patients with FGN but was absent in the biopsy specimens of 31 patients without FGN [12].

These studies showed that DNAJB9 is one of the most abundant glomerular proteins in FGN patients. Its absence in biopsies from patients with amyloidosis, other GN, or healthy controls established DNAJB9 as the first biomarker for FGN, allowing differentiation from amyloidosis and other glomerular diseases [15,16]. The role of DNAJB9 in the pathogenesis of FGN remains to be fully elucidated, and further research is needed to determine whether it plays a causative role or is merely a marker of the disease [17].

As current treatment options remain largely empirical with variable efficacy, understanding the molecular mechanisms underlying FGN is critical for the development of targeted therapies [3].

As FGN is an uncommon disorder of unclear etiology and low prevalence, we aimed to present our clinical experience of FGN from a tertiary nephrology center combined with the expertise and knowledge of a referral pathology team. The study will attempt to integrate our current experience to address gaps in the understanding of various aspects of the disease, improve management of the disease, and contribute to data in the field. It is also hoped that the study will identify future directions for the unclear pathophysiology.

## 2. Methods

In this retrospective cohort study, a total of eleven patients (six female and five male) diagnosed with fibrillary glomerulonephritis (FGN) at the Nephrology Clinic of G. Gennimatas Hospital between 2016 and 2025 were evaluated. The study was approved by the Medical Scientific Review Board of GNA “G. Gennimatas” (ethics approval number 369; date of approval 5 January 2024).

### 2.1. Patients

Patients Inclusion criteria:

Patients who met the diagnostic criteria for FGN were included in the study. No exclusion criteria were applied regarding comorbidities (e.g., DM, MGUS, cancer), as these conditions do not preclude the diagnosis [5,7].

The diagnosis of FGN is documented by the presence of at least one of the following criteria in renal biopsy specimens [13,14].

1. Deposition of fibrils in the glomerulus by light electron microscopy. The fibrils must have the following characteristics:
  - Random arrangement;
  - Diameter of 14 to 24 nm;
  - Deposition in the mesangium and the glomerular basement membrane.
2. Expression of DNAJB9 in immunohistochemistry

Eleven patients were included. Patient data extracted from medical records included demographic data [sex, race, age at diagnosis, follow-up period], and clinical data included the clinical presentation of the disease, including the presence of hematuria on urinalysis, proteinuria (urinary protein excretion > 300 mg/day), nephrotic range proteinuria (>3.5 g/day), and hypertension, defined as systolic blood pressure > 140 mmHg, diastolic blood pressure > 90 mmHg, or the use of antihypertensive medication (KDIGO 2021) [5,10,11,18]. A medical history was taken, focusing on possible comorbidities related to FGN, such as MGUS [18], diabetes mellitus, cancer, and autoimmune diseases.

Laboratory data collected at the time of diagnosis included the following: blood test values for urea, creatinine, albumin, and total serum protein; urine test results (24-h urine protein excretion and urinalysis); immunological markers; ANCA, ANA, and anti-dsDNA antibodies; serum and urine protein immunoelectrophoresis; and screening for hepatitis B, hepatitis C, and HIV. Follow-up laboratory data included final recorded values for urea, creatinine, and 24 h urine protein excretion.

The renal biopsy findings which were subjected to assessment included the following: the histological pattern, as revealed by light microscopy; the degree of glomerulosclerosis, as determined by light microscopy; the degree of interstitial fibrosis and tubular atrophy, as determined by light microscopy; the results of immunofluorescence analysis; fibril morphology, as determined by electron microscopy; the expression of DNAJB9; and Congo Red staining. All biopsy specimens were evaluated at a single center by the same group of renal pathologists, to ensure consistency and minimize interobserver variability.

Each patient’s treatment regimen and response to therapy were documented using renal function markers. Key indicators for the evaluation of treatment response included serum creatinine and proteinuria. Renal dysfunction was defined as serum creatinine > 1.2 mg/dL and proteinuria as urinary protein excretion > 300 mg over 24 h [17].

Therapeutic response was classified as follows:

- Complete response (CR): proteinuria < 0.5 g/day and normal renal function (serum creatinine < 1.2 mg/dL);
- Partial response (PR): reduction in proteinuria by >50% from the peak recorded value, stable renal function;
- Persistent renal dysfunction (PRD): failure to meet the criteria for CR or PR or worsening renal function without progression to ESRD;
- ESRD: eGFR < 15 mL/min.

As there are no established guidelines for treatment response in FGN, we defined outcomes using criteria from previously published series [5,10,11].

### 2.2. Statistics

The data were summarized using descriptive statistics. Continuous variables were expressed as means and standard deviations, with additional measures such as medians, ranges, and interquartile ranges reported as appropriate. The distribution of each variable was assessed using the Shapiro–Wilk and Kolmogorov–Smirnov tests for normality. Non-parametric tests were used to compare paired measurements. Specifically, the Wilcoxon signed ranks test was applied to assess differences between initial diagnosis and subsequent follow-up. All analyses were two-tailed, with a significance level set at *p* < 0.05.

Data analysis was performed using IBM SPSS Statistics 17.0.

## 3. Results

### 3.1. Demographic, Clinical Features and Laboratory Data

The study included eleven patients, six females and five males, with a median age at diagnosis of 58 years (IQR: 50.5–62). All patients had proteinuria at renal biopsy, with a median 24 h urine protein of 3.70 g (IQR: 3.2–5 g, mean: 4.61 g, SD: 2.93 g) and a median serum creatinine of 1.6 mg/dL (IQR: 1–2 mg/dL, mean: 1.89 mg/dL, SD: 1.22 mg/dL). At the time of diagnosis, nine patients (81%) had nephrotic range proteinuria without full-blown nephrotic syndrome, and eight patients had microscopic hematuria. Three patients had macroscopic hematuria without hypoalbuminemia, and one patient had peripheral oedema. Hyperlipidemia was observed in six patients.

Regarding the presence of comorbidities in the FGN patients, one patient had been diagnosed with systemic lupus erythematosus (SLE), one with sarcoidosis, and another with rheumatological symptoms without a confirmed diagnosis. Four patients were diagnosed with hypothyroidism, and one patient was diagnosed with lung cancer two months before the diagnosis of FGN. All patients tested negative for HIV, HBV, and HCV, and imaging studies were also negative.

Table 1 shows the demographic and clinical characteristics of patients with a diagnosis of FGN confirmed by renal biopsy. It should be noted that two patients were diagnosed after repeat biopsy.

All patients were tested for paraproteinemia. In one patient (patient 8), a bone marrow biopsy was performed because of the predominance of λ light chains on immunofluorescence despite negative serum and urine immunoelectrophoresis. The results showed a polyclonal plasma cell population with a κ/λ ratio of approximately 5/2. This finding is consistent with a diagnosis of plasma cell dyscrasia type MGUS [18,19]. Six of the eleven patients were ANA positive.

### 3.2. Pathology Findings

**Figure 1:**
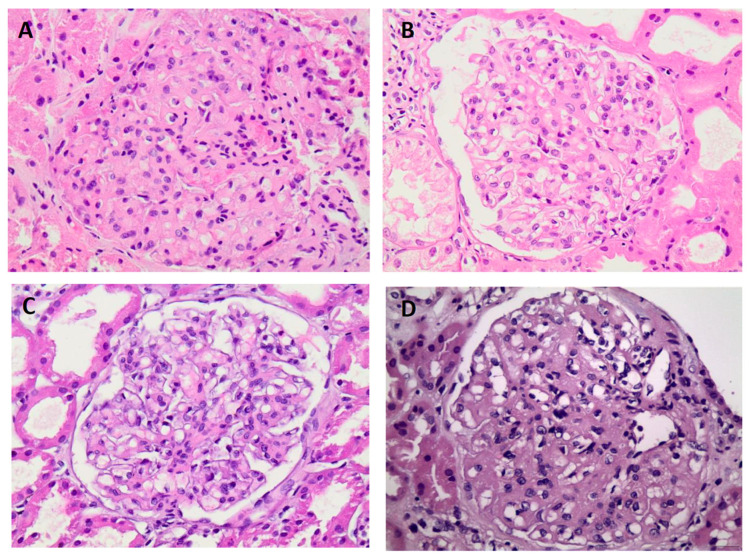
Patterns on light microscopy. (**A**) Diffuse sclerosing pattern (patient 1—H&Ε ×400). (**B**) Mesangial proliferative pattern (patient 4—H&Ε ×400). (**C**) Mesangial proliferative pattern (patient 5—H&Ε ×400). (**D**) Membranous pattern (patient 7—H&Ε ×400).

Renal biopsy results showed that six patients had a mesangial proliferative pattern under light microscopy. Two patients had crescents and necrosis involving at least 50% of the glomeruli, consistent with crescentic glomerulonephritis. Another patient had sclerosing changes, another had a membranoproliferative pattern, another had a membranous pattern, and one patient had both mesangial hyperplasia and a membranoproliferative pattern, Figure 1. The mean percentage of globally sclerotic glomeruli was 37%. Crescents were reported in three cases, with interstitial fibrosis and tubular atrophy ranging from 20% to 35% (avg. 26%). Congo Red staining was negative.

Immunofluorescence results showed that 10 out of 11 cases were positive for IgG, with a mean intensity of 1.6+ (on a scale of 0–3+). The positivity was mainly observed in the mesangium and glomerular basement membrane and manifested as a granular or linear pattern. In addition, 27% of cases were positive for IgA (mean intensity 1.5+), while 45% were positive for IgM (mean intensity 1.5+). Glomerular C3 deposition was detected in 10 out of 11 patients (mean intensity 2.3+), while C1q was found in only 2 patients (mean intensity 1.5+). Glomerular deposits were positive for κ (three patients) and λ chains (six patients). Three patients had both light chains, and three others had only the λ chain. Further testing (SPEP/IFE) revealed monoclonal IgG-λ in one patient.

**Figure 2:**
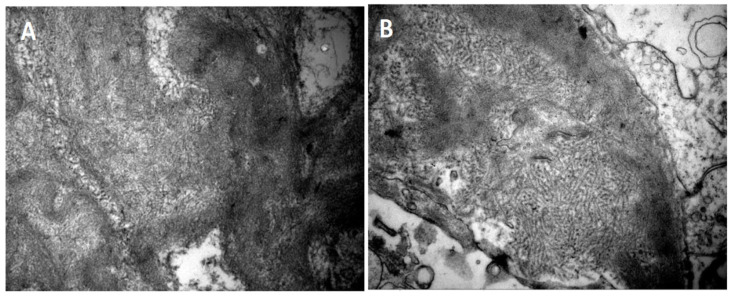
Electron microscopy findings in FGN. (**A**) Fibril deposits with random orientation in the mesangium (patient 4—uranyl acetate ×28,000). (**B**) Fibril deposits in the mesangium and glomerular membranes (patient 1—uranyl acetate ×18,000).

Electron microscopy was performed on 10 of the 11 renal biopsies. The fibrils showed a random orientation, with a linear and unbranched configuration, an average size of 10 to 20 nanometers, and a predominant distribution within the mesangium and glomerular basement membrane, Figure 2. Fusion of the foot processes was observed in 8 of the 10 cases examined by electron microscopy.

**Figure 3:**
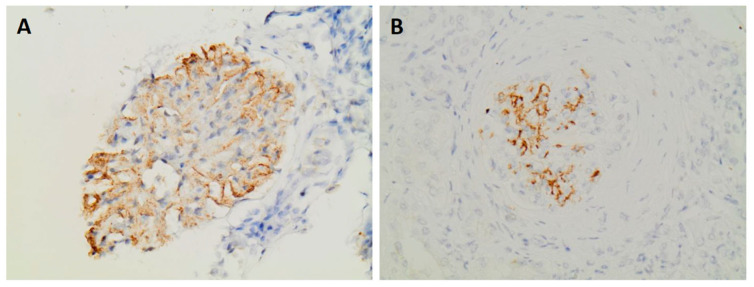
Glomeruli showing positive staining for DNAJB9 (**A**) Patient 1—DNAJB9 ×400. (**Β**) Patient 4—DNAJB9 ×400.

Immunohistochemistry for DNAJB9 was performed in five patients and showed intense positivity in the mesangium and in two cases also in the glomerular basement membrane, Figure 3. In one patient (patient 3), the diagnosis of FGN was made on the basis of positivity for the marker, as electron microscopy was not available.

The histological findings for each patient are summarized in Table 2.

### 3.3. Treatment and Clinical Outcome

All patients received a renin–angiotensin–aldosterone axis inhibitor and immunosuppression.

Table 3 summarizes the therapeutic regimens and clinical responses of the patients. One patient was initially diagnosed with lupus nephritis and was diagnosed with FGN on repeat renal biopsy. Immunosuppressive therapy (glucocorticoids, cyclophosphamide and MMF) was administered for the treatment of lupus nephritis, which was subsequently discontinued following the diagnosis of FGN. The majority of patients were treated with corticosteroids for six months, along with two doses of rituximab at 1 g (1 g) administered intravenously at 15-day intervals. A repeat dose of rituximab (1 g) was given six months later. One patient (patient 2) received cyclophosphamide because of uncontrolled diabetes, while two patients (patients 10 and 11) received intravenous cyclophosphamide (15 mg/kg) and rituximab because of rapidly progressive glomerulonephritis and the presence of crescents in ≥50% of the glomeruli. In another patient (patient 9), non-response to multiple repeated cycles of rituximab led to a change in treatment to mycophenolate mofetil (MMF-2 g daily). Treatment decisions were primarily guided by disease progression, kidney function, and the presence of crescents on biopsy, with adjustments made in response to therapeutic outcomes.

The median duration of follow-up was 24 months (interquartile range: 9–42 months). Based on predefined criteria, eight patients (73%) achieved a partial remission, defined as a >50% reduction in proteinuria from the peak recorded value with stable renal function. One patient (9%) met the criteria for complete remission, characterized by proteinuria < 0.5 g/day and serum creatinine < 1.2 mg/dL. Two patients (18%) exhibited persistent renal dysfunction, defined as failure to achieve partial or complete remission or worsening renal function without progression to ESRD. No patients progressed to ESRD during the follow-up period.

In this study, the majority (eight out of eleven patients, 80%) of patients showed a reduction in proteinuria of more than 50% with stable renal function and partial remission. One patient (patient 5) showed complete remission after a total of four doses of rituximab administered every six months, resulting in normal renal function without proteinuria or hematuria. A significant decrease in proteinuria was observed after the first dose of rituximab (from 590 mg to 2.9 g/24 h).

Two patients (8 and 9) had persistent renal dysfunction. One of these patients died from complications of SARS-CoV-2 infection, resulting in a mortality rate of 9.1%, while the other showed a significant reduction in proteinuria after treatment with both rituximab and MMF. However, due to an allergic reaction to rituximab and gastrointestinal discomfort from MMF, the patient discontinued immunosuppressive treatment, resulting in further worsening of proteinuria.

The response to each treatment regimen is variable, with the majority of cases showing a significant reduction in proteinuria. The Wilcoxon signed ranks test confirmed this improvement, revealing a statistically significant decrease of proteinuria from diagnosis to follow-up (mean Upr: 4.6 to 1.9 mg/g; Z = −2.758, *p* = 0.006). In contrast, serum creatinine levels remained stable, with no significant difference observed between the two time points (mean sCr: 1.4 mg/dL at both diagnosis and follow-up; Z = −1.192, *p* = 0.233). This stability should not be interpreted as a lack of response, but rather as the preservation of renal function. Moreover, univariate analysis using the Mann–Whitney U test showed no significant difference in proteinuria at diagnosis between patients with compete/partial remission (favorable outcome) and patients with persistent renal dysfunction (unfavorable outcome), (U = 9.000, *p* = 1.000). Given the small sample size and lack of significance, these results should be interpreted with caution.

## 4. Discussion

Eleven patients diagnosed with FGN at our center between 2016 and 2025 are included in this study. The clinical and histological characteristics appear to be consistent with those described in large retrospective studies. Most patients had nephrotic range proteinuria without nephrotic syndrome at diagnosis, and the mesangial proliferative histological pattern predominated in patients.

Although FGN remains a rare and heterogeneous disease, our findings, in line with previous reports, highlight the need for stronger prognostic markers. Factors such as proteinuria and serum creatinine at diagnosis and the presence of glomerular crescents in kidney biopsy, may significantly influence long-term outcomes [5,20]. In our cohort, univariate analysis did not demonstrate a significant relationship between proteinuria at diagnosis and therapeutic outcomes (U = 9.000, *p* = 1.000). These findings should be interpreted with caution, given the small sample size. Despite this, previous studies have demonstrated that nephrotic range proteinuria and impaired renal function at diagnosis are associated with progression to ESRD [21].

Currently, there is no preferred treatment or established therapeutic protocol for the treatment of FGN, and there are no randomized clinical trials to guide therapeutic decisions [22]. Given the potential autoimmune nature of FGN, rituximab, a monoclonal antibody targeting CD20 on B lymphocytes, has been tested and is considered one of the main therapeutic options for FGN. The first and only prospective study on the use of rituximab in FGN patients was conducted by Erickson et al. in 2021 [23]. Drugs such as corticosteroids, cyclophosphamide, cyclosporine, mycophenolate, azathioprine, lenalidomide, and sirolimus have also been shown to have no significant therapeutic benefit [5,10,22].

Until recently, the diagnosis of FGN was based on electron microscopy. However, this approach has evolved significantly since the identification of the DNAJB9 marker [15,24]. Despite the diagnostic limitations imposed by the inability to use electron microscopy, FGN was diagnosed using DNAJB9 staining. In a large clinical study that included biopsies from 84 patients with FGN [14], 21 with amyloidosis, 98 with other glomerular diseases, and 11 healthy controls, staining for DNAJB9 was found to have a sensitivity of 98% and a specificity of 99% for the diagnosis of FGN. Recent studies had shown that the positive expression of the DNAJB9 marker is considered to be highly specific for FGN, as further data emerged [15,16].

DNAJB9, also known as ERdj4 or Mdg-1, is a member of the DNAJ protein family, which acts as a co-chaperone for the HSP70 heat shock protein family [25]. DNAJB9 assists in protein folding and degradation within the endoplasmic reticulum and is found at low levels in most body cells, with higher expression in tissues such as the liver and placenta [26,27]. In the kidney, DNAJB9 is present in tubular cells, podocytes, epithelial cells, and endothelial cells [27].

The role of DNAJB9 in the pathogenesis of FGN is still under investigation [27]. It has been hypothesized that DNAJB9 acts as an autoantigen, triggering an autoimmune response when misfolded proteins accumulate in the glomerulus [12]. However, no circulating autoantibodies to DNAJB9 have been detected. An alternative hypothesis is that DNAJB9 binds to misfolded IgG and contributes to fibril formation [27]. Despite these hypotheses, many questions remain, including the formation of fibrils and why immunosuppressive therapy is not as successful.

Possible next steps in understanding the pathogenesis of FGN include the study of circulating levels of DNAJB9 and its antibodies and the possible development of animal models. Measuring the plasma levels of DNAJB9 had been performed, according to which patients with FGN have higher levels of DNAJB9 compared to controls [26], possibly due to increased production and decreased clearance [26]. However, before potential clinical use, these initial findings need to be confirmed, studies in patients with preserved renal function and the development of simpler techniques.

This study described two rare cases of FGN associated with sarcoidosis and SLE. The rarity of documented cases of these co-morbidities in the available literature supports the hypothesis that these diseases and FGN are related, and further research is recommended. One case involves a woman diagnosed with lupus nephritis and FGN. Fibrils in patients with FGN associated with SLE are significantly smaller, ranging in size from 8 to 15 nm, and may have a “fingerprint” pattern. A retrospective study of 185 lupus nephritis biopsies found these deposits in 17.3% of patients, but only 1% had fibrils resembling FGN [28]. The association of FGN with SLE has been sparsely documented in the literature. Examples included a 12-year-old with class IV lupus nephritis who was diagnosed with FGN on repeat biopsy [29], a 28-year-old man with SLE and thrombotic microangiopathy [TMA] who was also diagnosed with FGN [30], and in a study of 66 FGN patients, two cases of SLE but no histologically confirmed lupus nephritis [5]. Rosenstock et al. reported one case of SLE in 67 FGN patients, none of whom had features of lupus nephritis [2]. A recently published case report described a woman who has had SLE for 20 years. The patient presented with proteinuria and a biopsy revealed FGN with DNAJB9 positivity [31]. Consequently, the association between GN and SLE remains poorly documented, with limited data available on their clinical course, therapeutic interventions, and prognosis.

In addition, one patient was diagnosed with sarcoidosis immediately after the diagnosis of FGN and during the investigation of secondary causes. Kidney damage in patients with sarcoidosis is rare [10–20%] and is usually the result of calcium homeostasis disorders, manifesting as nephrolithiasis and nephrocalcinosis, and the rarer granulomatous tubulointerstitial nephritis. The literature on glomerular damage in patients with sarcoidosis is limited, and the pattern of glomerular damage is variable [32]. Cases of sarcoidosis associated with membranous nephropathy, minimal change disease, focal segmental glomerulosclerosis, and IgA nephropathy have been documented [32].

The present study is not without its limitations. The study is of a single-center nature, which may potentially introduce selection bias, with a limited number of patients, which limits the statistical power and impacts the generalizability of the findings. It is important to note, however, that the disease under investigation is rare and the heterogeneity in clinical presentation and treatment responses complicate the ability to draw definitive conclusions. The objective is to engage in multicenter studies. Our cohort included one patient with SLE and one with lung cancer. These conditions could potentially influence renal pathology and disease outcomes. These factors should be considered when interpreting the results.

The association of FGN with sarcoidosis is rare, with only one documented case in the literature to date. This particular study was retrospective and looked at cases of FGN associated with the expression of the DNAJB9 marker. The study reported two cases of sarcoidosis associated with FGN, and these cases were in the context of autoimmune diseases that may be associated with FGN [7].

## 5. Conclusions

FGN is a rare glomerular disease that presents with a variety of clinical and histological features, making the differential diagnosis complex. The identification of DNAJB9 as a precise and specific marker for FGN has fundamentally altered the diagnostic process for the disease, thereby decreasing the reliance on electron microscopy. Despite these advances, effective treatment options for FGN remain limited. Our experience as a tertiary nephrology center highlights the evolving landscape of FGN management and emphasizes the importance of individualized treatment strategies.

## Figures and Tables

**Table 1:** Demographic and clinical characteristics at biopsy. DM2: type 2 diabetes mellitus, AF: atrial fibrillation, CKD: chronic kidney disease, SLE: systemic lupus erythematosus.

Patients	Gender	Age	Serum Creatinine [mg/dL]	Proteinuria [gr/24 h]	Hematuria	Hypertension	Comorbidities
1	Male	45	1.7	3.70	−	+	-
2	Female	58	1.4	3.50	−	+	DM2, rheumatologic disease
3	Female	31	0.9	3.00	+	+	Hypothyroidism, tonsillectomy
4	Female	69	1.7	4.50	+	+	DM2, hypothyroidism, AF, CKD, smoking
5	Male	34	0.8	1.33	+	−	Smoking
6	Female	60	2.4	3.78	+	−	Thyroid nodules, dyslipidemia, SLE
7	Male	63	1.2	11.68	+	+	Obesity, smoking
8	Male	61	1.6	8.00	+	+	Colon polyps
9	Female	56	0.8	1.80	−	−	Hypothyroidism, dyslipidemia, sarcoidosis
10	Male	74	4.5	5.60	+	+	Hypothyroidism, CKD
11	Female	57	3.6	4.00	+	−	Lung cancer, dyslipidemia

**Table 2:** Histological findings (NA: not applicable).

Patients	Histological Pattern	Glomerulosclerosis	Tubular Atrophy/Interstitial Fibrosis	Crescents	DNAJB9	Immunofluorescence
1	Diffuse sclerosing	75%	35%	−	NA	IgG 1+, IgM 1+, IgA 1+, C3 3+, C1q trace, κ negative, λ 1–2 +
2	Membranoproliferative	66%	25–30%	−	NA	IgG 1–2+, IgM 1–2+, IgA trace, C3 1–2+, C1q trace, κ λ negative
3	Mesangial proliferative	30%	25%	−	+	IgG 2+, IgM 2–3+, IgA trace, C3 3–4+, C1q, κ, λ trace
4	Mesangial proliferative	63%	35%	−	+	IgG 1–2 +, IgM trace, ΙgA negative, C3 1–2+, C1q negative, λ 1–2+, κ negative
5	Mesangial proliferative	18%	20%	1 cellular	NA	IgG 2–3+, IgM trace, ΙgA 2–3+, C3 2+, C1q 2+, λ 3+, κ 2+
6	Mesangial proliferative + membranoproliferative	18%	25%	−	NA	IgG 1–2+, IgM 1–2+ IgA negative, C3 3+, C1q, κ, λ trace
7	Membranous	60%	25%	−	+	IgG 1–2+, IgM 1+, IgA trace, C3 2–3+, C1q trace, κ trace, λ 2+
8	Mesangial proliferative	40%	20%	−	NA	IgG 1+, IgM trace – 1+, IgA negative, C3 2–3+, C1q 1+, κ 1+, λ 2–3+
9	Mesangial proliferative	6%	20%	−	NA	IgG 2–3+, IgM trace, IgA 1+, C3 2+, C1q trace, κ 1–2+, λ 3+
10	Crescentic	27%	35%	4 [2 cellular, 2 fibrocellular]	+	IgG 1+, IgM trace, IgA negative, C3 trace, C1q negative, κ, λ trace
11	Crescentic	8%	25%	8 [5 cellular, 3 fibrocellular]	+	IgG, IgA negative, IgM trace, C3 1–2+, C1q trace, κ, λ negative

**Table 3:** Treatment and therapeutic response. GC: glucocorticoids, RTX: rituximab, CYC: cyclophosphamide, MMF: mycophenolate mofetil, PR: partial remission, CR: complete remission, PRD: persistent renal dysfunction, sCr: serum creatinine, Upr: urinary protein.

Patients	Follow up Period [Months]	Immunosuppression	sCr [mg/dL] Diagnosis	sCr [mg/dL] Last Measurement	Upr [g/24 h] Diagnosis	Upr [g/24 h] Last Measurement	Therapeutic Response
1	24	GC + RTX	1.7	1.7	3.7	0.8	PR
2	24	CYC + RTX	1.4	1.5	3.5	0.5	PR
3	6	GC + RTX	0.9	0.9	3	1.2	PR
4	12	GC + RTX	1.7	1.4	4.5	2.7	PR
5	48	GC + RTX	0.8	1	1.3	0.1	CR
6	36	GC + CYC + MMF	2.4	1.7	3.7	0.05	PR
7	12	GC + RTX	1.2	1.2	11.6	1.9	PR
8	60	GC + RTX	1.6	2.2	8	7.8	PRD
9	72	GC + RTX + MMF	0.8	0.7	1.8	2.5	PRD
10	6	GC + CYC + RTX	4.5	3.3	5.6	1.6	PR
11	2	GC + CYC + RTX	3.8	1.6	4	1.5	PR

## Data Availability

Upon request, the corresponding author can provide the datasets used and analyzed in this study.
